# Integrating Metaecosystem Framework with Ecosystem Service Dynamics in Sociohydrosystems

**DOI:** 10.1093/biosci/biaf192

**Published:** 2025-12-22

**Authors:** Amélie Truchy, David Eme, Franck Jabot, Fabrice Vinatier, Aurélien Jamoneau, Eric J Petit, Bertrand Villeneuve, Laure Carassou, Thibault Datry, Alienor Jeliazkov

**Affiliations:** INRAE, UR RiverLy, Centre Lyon-Grenoble Auvergne-Rhône-Alpes, 5 rue de la Doua CS70077, 69626 Villeurbanne Cedex, France; Department of Aquatic Sciences and Assessment, Swedish University of Agricultural Sciences, 750 07 Uppsala, Sweden; INRAE, UR RiverLy, Centre Lyon-Grenoble Auvergne-Rhône-Alpes, 5 rue de la Doua CS70077, 69626 Villeurbanne Cedex, France; INRAE, VetAgro Sup, Université Clermont Auvergne, UMR Ecosystème Prairial, 63000 Clermont-Ferrand, France; LISAH, Univ Montpellier, AgroParisTech, INRAE, Institut Agro, IRD, 34060 Montpellier, France; INRAE, UR EABX, 50 avenue de Verdun, 33612 Cestas Cedex, France; DECOD (Ecosystem Dynamics and Sustainability), Institut Agro, Ifremer, INRAE, 35000 Rennes, France; INRAE, UR EABX, 50 avenue de Verdun, 33612 Cestas Cedex, France; INRAE, UR EABX, 50 avenue de Verdun, 33612 Cestas Cedex, France; INRAE, UR RiverLy, Centre Lyon-Grenoble Auvergne-Rhône-Alpes, 5 rue de la Doua CS70077, 69626 Villeurbanne Cedex, France; University of Paris-Saclay, INRAE, HYCAR, 1 rue Pierre-Gilles de Gennes, 92160 Antony, France

**Keywords:** biodiversity, metacommunity, metapopulation, management, landscape

## Abstract

Sociohydrosystems provide vital ecosystem services such as water purification, flood regulation, and climate regulation. However, understanding the complex relationships among ecosystem processes, hydrosystem dynamics, and ecosystem services is crucial. The metaecosystem framework, which is focused on the flows of organisms, matter, and energy among ecosystems across spatial and temporal scales, provides valuable tools for more holistic ecosystem assessments. The present article shows that a unified framework is crucial for understanding how ecosystem services are influenced by spatiotemporal dynamics of organisms, matter, and energy, advancing our understanding of how changes in one area of the hydrosystem translate into changes in other areas. The proposed framework also helps identify synergies and trade-offs among ecosystem services and the influence of environmental conditions. Finally, this unified framework enhances our ability to inform decision-making; design adaptive management strategies that consider changing environmental conditions, especially in the context of climate change; and mitigate social inequalities.

Aquatic continental ecosystems are among the most threatened ecosystems on Earth and undergo high rates of degradation (Reid et al. 2019, Albert et al. 2021). However, they are crucial to humanity because they contribute to human well-being and provide an array of ecosystem services (see box 1; Daily 1997). Ecosystem services encompass drinking and irrigation water, food, transport route, hydropower, cooling nuclear power plant, and recreational activities, for example (MEA 2005, Haines-Young and Potschin 2018). The concept of sociohydrosystems was therefore developed to explicitly account for strong interactions between humans and continental aquatic ecosystems (Sivapalan et al. 2012, 2014). Sociohydrosystems are indeed an interrelated set of natural habitats, resources, actors, institutions, and infrastructures recognizing the role played by aquatic ecosystems for humans, as well as being the target of different policies aiming at mitigating human impacts (e.g., WFD 2000, Sivapalan et al. 2012).

Box 1. **Glossary.**

**Benefit:** positive change in people’s well-being (Tallis et al. 2012) as a result of ecosystem services provision. Changes in well-being include materials essential for life and contributions to health, security, social relations, and freedom of choice and actions (MEA 2005).

**Cascade model:** a model where ecosystems are defined by properties (i.e., biophysical structures and ecosystem processes) that produce ecosystem functions, which in turn support ecosystem services that benefit humans, and to which a value (e.g., health or monetary) can be attributed (Haines-Young and Potschin 2010).

**Disservice:** “the negative effects of nature on human well-being” (Blanco et al. 2019).

**Ecosystem functioning:** the joint effects of all processes that sustain an ecosystem (Reiss et al. 2009).

**Ecosystem process:** a process emerges at the ecosystem level and involves interactions between species and their environment, often involving transformation of nutrients and energy, generation of habitat structures or maintenance of populations (Truchy et al. 2015). Ecosystem function is often used as a synonym (Reiss et al. 2009).

**Ecosystem services:** the conditions and processes through which ecosystems, and the species that make them up, sustain and fulfill human life (Daily 1997).

**Ecosystem services bundles:** sets of ecosystem services that co-occur at a specific location and at a particular time (Saidi and Spray 2018).

**Ecosystem services demand:** the level of service provision desired or required by societies, driven by human needs, preferences, values, institutions, market prices, and technology (Villamagna et al. 2013).

**Ecosystem services flow:** processes that actually connect supply and demand in space and time (e.g., flows of people, organisms or matter) within the concept of the “service provision chain” (Metzger et al. 2021).

**Ecosystem services provision:** the delivery of a service to be used or enjoyed by people (Villamagna et al. 2013) when the supply meets the demand.

**Ecosystem services supply:** the capacity of ecosystem functions and biophysical elements in an ecosystem to provide a given ecosystem service, whether or not humans recognize, use, or value that service (Tallis et al. 2012, Burkhard and Maes 2017).

**Ecosystem services synergies:** ecosystem service synergies arise when multiple services are enhanced simultaneously (Raudsepp-Hearne et al. 2010).

**Ecosystem services trade-off:** Ecosystem services trade-offs arise when the provision of one ecosystem service is enhanced at the cost of the production of reducing the provision of another service (Raudsepp-Hearne et al. 2010).

**Extinction debt:** the time delay to loose species following habitat destruction (Tilman et al. 1994).

**Immigration credit:** the time delay for species immigrating following a forcing event (Jackson and Sax 2010).

**Metapopulation:** a set of local populations of a single species that are linked by dispersal (Hanski 1998).

**Metacommunity:** “a set of local communities that are linked by dispersal of multiple potentially interacting species” (Leibold et al. 2004).

**Metaecosystem:** “a set of ecosystems connected by spatial flows of energy, materials and organisms, across ecosystem boundaries” (Loreau et al. 2003).

**Scale:** the grain of a variable indexed by time or space (Wiens 1989).

**Service provision chain:** Ecosystem service provision is the result of interactions between different components (Fisher 2009 et al. 2009, Tallis et al. 2012). These components include the service provision and demand areas connected by ecosystem services flows across the service-connecting area.

**Sociohydrosystem:** a set of habitats, resources, actors, institutions and infrastructures recognizing both the role played by water ecosystems for humans, as well as being the target of different policies aiming at mitigating human impacts (e.g., WFD 2000).

The pressures threatening sociohydrosystems and their subsequent ecosystem services are multiple (with interactive effects on sociohydrosystems) and apply at multiple scales (Parmesan and Yohe 2003, Vörösmarty et al. 2010, Grill et al. 2019, Reid et al. 2019). Pressures can be global (climate change) or local (water abstraction, geomorphological alteration) or can span various scales depending on context (contamination, habitat loss or degradation). The effects of a pressure can propagate spatially through the sociohydrosystems (e.g., Villeneuve et al. 2018), affecting locally produced ecosystem services, and ecosystem service benefits may be appreciated at locations kilometers away or temporally delayed from the ecosystem services production sites (Fisher et al. 2009). Spatial lags occur, for instance, when pollution takes place in headwaters but affects drinking water quality in downstream cities. Carbon sequestration also takes place locally in peatlands, wetlands, or alluvial forests, but the climatic effects are global (e.g., Ribeiro et al. 2021). Besides spatial lags, time lags also exist—for example, in karst aquifers that transit large volumes of water from infiltration zones to surface springs several months after rainfall (Martin-Rodriguez et al. 2023). This hierarchy of spatiotemporal scales calls for the development of management strategies that take into account the existing interactions among multiple organizational levels and scales and, in particular, the fluxes linking these specific scales (Peters et al. 2007).

Historically, ecosystem services frameworks have been focused on defining ecosystem service provision (mostly by natural sciences; Assis et al. 2023) or ecosystem service demand (mostly by social sciences; Peter et al. 2022, Assis et al. 2023), ignoring their inherent spatiotemporal dimensions and dynamics. Later on, integrating ecosystem services into conservation planning favored frameworks considering ecosystem services spatial dimensions (Wilbanks 2006), such as the service provision chain (figure 1; Fisher et al. 2009). Indeed, ecosystem services provision arises from the interactions between the service-provisioning area and the service-benefiting area connected by biotic and abiotic flows through the service-connecting area. Service-provisioning and service-benefiting areas can spatially co-occur or be apart. In case of a spatial mismatch, service provisioning would require geophysical, ecological, cultural, or economic flows (Assis et al. 2023) that connect the service-provisioning and service-benefiting areas (Fisher et al. 2009). These flows can encompass organism movements (e.g., aquatic pollinating insects flying from water to crops; Raitif et al. 2019) or raw material (i.e., resources) transport. In particular cases, ecosystem services provision only occurs when interrupting or reducing flows between service-provisioning and service-benefiting areas, as when most of water flows is stored in reservoirs to protect downstream cities against floods (Metzger et al. 2021). Although the conceptualization of the service provision chain is gaining momentum (Burkhard and Maes 2017, Winkler et al. 2021), it currently lacks an explicit link with the existing spatialized frameworks of ecology (but see Le Provost et al. 2023, Mitchell et al. 2024).

**Figure 1.**
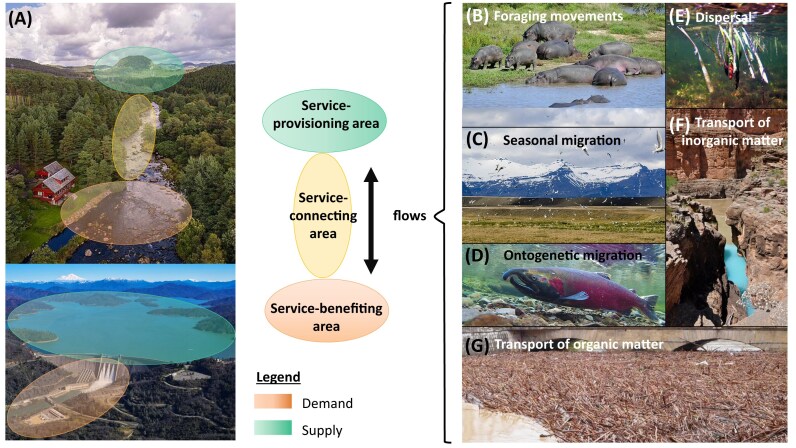
Service provisioning chain and its inherent flows. Service provisioning chain (Fisher et al. 2009) can be applied to multiple sociohydrosystems (a) as service benefiting areas (service-benefiting area; the orange lower circles) and service provisioning areas (service-provisioning area; the green top circles) can easily be identified. Service-benefiting and service-provisioning areas can overlap. When not overlapping, service-provisioning and service-benefiting areas are linked by flows of organisms, matter and energy (b)–(g) occurring in service-connecting areas (the yellow middle circle). Organism flows can encompass foraging movements (b) (e.g., grazing of hippopotamuses on riparian grasslands), seasonal (c) (e.g., arctic terns migrating from Antarctica to Iceland) and ontogenetic migrations (d) (e.g., salmons migrating from headwaters to sea and vice versa to complete their life cycles), and dispersal (e) (of plant propagules during floods). Composition and configuration of the sociohydrosystems can directly influence flows of organisms, energy and matter between service-provisioning and service-benefiting areas (e.g., a dam blocking fish migration). Likewise, the needs and preferences of beneficiaries can shape societal demand for a particular ecosystem service and impact flow from a service-benefiting area to a service-provisioning area. Photographs: (a) Jules Verne Times Two/CC-BY-SA 4.0 and Bureau of Reclamation/CC BY-NC-SA 2.0, (b) B. Dupont/CC-BY-SA 2.0, (c) Jakub Fryš/CC-BY-SA 4.0, (e) BLMOregon/CC BY 2.0, (e) Phil’s 1stPix/CC BY-NC-SA 2.0, (f) Grand Canyon National Park/CC BY 2.0, (g) A. Truchy

Indeed, spatialized ecosystem service frameworks developed independently from the paradigm shift that ecology experienced, recognizing the importance of spatial flows of individuals, matter, and energy in the functioning of ecosystems (e.g., Loreau et al. 2003, Gounand et al. 2018a, Cid et al. 2022). The metaecosystem concept posits that processes at the local and regional scales shape the dynamics of biodiversity and the environmental conditions of a landscape (Gounand et al. 2018a). Regional-scale processes are characterized by flows of individuals of a species, energy, and matter among local populations, communities, and ecosystems, whereas local-scale processes usually encompass responses to habitat conditions and interactions among organisms (Leibold et al. 2004, Gounand et al. 2018a). Local populations, communities, and ecosystems connected at the regional scale form, respectively, metapopulations, metacommunities, and metaecosystems (Hanski 1998, Loreau et al. 2003, Leibold et al. 2004, Massol et al. 2011). To date, a unified framework taking into account the spatiotemporal dynamics of the ecosystem processes (and processes interactions) underlying the spatiotemporal dimensions of ecosystem services production and supply is missing, limiting our ability to identify synergies and trade-offs between ecosystem services within a spatiotemporally explicit context and to understand how changes at a particular spatial or temporal scale translates into changes at other (larger or smaller) scales (e.g., spillover effects and telecoupling). In the present article, we therefore explore the potential contribution of metaecosystem approaches and their spatially explicit corollaries (Truchy et al. 2015, Scherer-Lorenzen et al. 2022) in the understanding and optimization of policy and management of ecosystem services in sociohydrosystems. We consider the whole variety of sociohydrosystems, including headwaters, rivers, lakes, wetlands, estuaries, and groundwaters, but also the terrestrial interface areas, such as riverbanks, riparian areas, and adjacent lands. We first summarize the spatiotemporal characteristics of sociohydrosystems that make them archetypical but also challenging templates of metaecosystem dynamics. In a second part, we explore the links between metaecosystem and ecosystem service theories in order to better understand ecosystem services maintenance and delivery in sociohydrosystems. In particular, we propose to disentangle the spatiotemporal links between the fundamental flows (e.g., organism movements and matter fluxes) within sociohydrosystems and the ecosystem services spatial dimension of the supply provision chain (i.e., service-provisioning area, service-connecting area, service-benefiting area). We then derive examples of how accounting for metasystem processes has a great potential to improve ecosystem services management and policy in the spatiotemporally explicit context of the sociohydrosystems. Finally, we draw a road map for future research that takes advantage of the metaecosystem approach for a better understanding of ecosystem services in sociohydrosystems, especially highlighting some conceptual, technical, and epistemological challenges.

## The spatiotemporal template of sociohydrosystems as metaecosystems

Flows of organisms and resources (i.e., organic and inorganic matter) across spatial scales structure and maintain local populations, communities, and ecosystems in sociohydrosystems (Truchy et al. 2015). In a metaecosystem perspective, two flow categories are distinguished: organism movements and passive resource flows (Gounand et al. 2018a). Organism movements are typically grouped in four main categories: foraging, seasonal migration, ontogenetic migration, and dispersal (figure 1b–g). Ontogenetic migration corresponds to a migration during which organisms undergo physiological modifications that make them change their preferential habitat (aquatic to terrestrial for many insects, fresh- to saltwater or inversely for migratory fishes). Historically, dispersal, which is “the tendency of organisms to live, compete and reproduce away from their birth place” (Massol et al. 2017), received a lot of attention in ecology from both a theoretical and empirical point of view. Dispersal plays a key role in the metapopulation framework (Hanski 1998), later extended to a multispecies context and giving rise to the metacommunity framework (Leibold et al. 2004).

At the population level, the dispersal of individuals among different populations may ensure rescue effects in otherwise declining populations (Granzotti et al. 2021) and may maintain the adaptive potential of these populations (Baguette et al. 2013). On the contrary, dispersal among isolated populations can weaken or impede local adaptation and can ultimately affect population persistence (Ronce 2007). At the community level, two major dispersal-related effects occur: mass effect and dispersal limitation. Mass effect describes the immigration of individuals of highly dispersive species from habitats in which they reproduce and survive best (i.e., sources) to suboptimal habitat patches (i.e., sinks), maintaining a certain level of taxonomic and functional diversity at the metacommunity level (Mouquet and Loreau 2003). In contrast, dispersal limitation prevents species from colonizing suitable habitats because of low dispersal abilities or abiotic or biotic barriers (Heino et al. 2015). Finally, at the ecosystem level, flows of individuals from different trophic levels from a habitat patch to another may change trophic interactions in the communities and stabilize or destabilize ecosystems (e.g., species coexistence, population asynchrony; Quévreux et al. 2023). If the metapopulation and metacommunity concepts are mostly focused on processes driving the maintenance of species and communities in fragmented landscapes, they lack a clear inclusion of other organism movements (e.g., foraging) driving the flow of matter within and among ecosystems that are, however, essential for ecosystem services.

The metaecosystem concept offers a larger perspective on organism movements by explicitly acknowledging foraging, seasonal, and ontogenetic migration as crucial processes behind ecosystem functioning and biodiversity resilience (Jacquet et al. 2022). Metaecosystems explicitly include passive flows of resources which are typically represented by the spatial flows of inorganic nutrients and organic matter (dissolved or particulate), including dead organisms considered to be nutrient sources (Gounand et al. 2018a, Scherer-Lorenzen et al. 2022). Passive resource flows occur through passive physical processes (e.g., runoff water, sedimentation) but also through organism movements (e.g., emerging aquatic insects providing high-quality nutrients for terrestrial ecosystems; Scherer-Lorenzen et al. 2022).

Within this metaecosystem framework, terrestrial areas adjacent to hydrosystems constitute reciprocal donor and recipient ecosystems connected through different pathways (i.e., the directionality of flows of organisms, energy, and matter) across ecosystem boundaries (Scherer-Lorenzen et al. 2022, Harvey et al. 2023). Some sociohydrosystems such as rivers and connected lakes have a peculiar spatiotemporal configuration linked to their dendritic structure and flow directionality that strongly constrains within-ecosystem flows of matter and energy (e.g., Wipfli and Baxter 2010, Heino et al. 2015, Cid et al. 2022). These flows are modulated by three spatial dimensions (Ward 1989): the longitudinal one, where upstream–downstream gradient determines flow direction, hydrological connectivity, and biotic zonation; the vertical one, where flows depend on the connectivity along the water column (i.e., links between pelagos and benthos in rivers and lakes) but also with soil, sediments through the hyporheic zone, and more generally with any groundwater (e.g., phreatic and karstic habitats); and the lateral one, where flows depend on the connectivity with adjacent aquatic and terrestrial ecosystems. These three spatial dimensions place sociohydrosystems at the center of flows of energy and matter between terrestrial and aquatic ecosystems and underpin the dominant role they play in ecosystem services delivery.

Time can be seen as a fourth dimension driving important sociohydrosystems processes (Stanford and Ward 1993). For instance, seasonality strongly conditions hydrodynamics, the dynamics of aquatic communities and ecosystem processes (e.g., Dai and Trenberth 2002, Caffrey et al. 2014, Sarremejane et al. 2021). In particular, community dynamics respond to abiotic changes that fluctuate over time and across different temporal scales—for example, from long-term geological time scale (e.g., glacial and interglacial oscillations during the Pleistocene; Hewitt 2000) or decadal trends (e.g., Alric et al. 2021, Tison-Rosebery et al. 2022) to between-year (e.g., Baranov et al. 2020, Ortega et al. 2021) and within-day variations (e.g., Rose et al. 2020). Communities also respond to extreme events, such as droughts and floods (Sarremejane et al. 2021). Furthermore, important time lags can be observed in nutrients dynamics because of complex mixing processes affecting residence time (Erostate et al. 2018) and in community responses to past environmental changes (Hamilton 2012, Rastetter et al. 2021), extinction debt, and immigration credit (Jackson and Sax 2010)—all potentially resulting in temporal or spatiotemporal mismatches between ecosystem services production and ecosystem services reception (Leadley 2010). A better integration of the metaecosystem framework into ecosystem services perspectives therefore requires a thorough knowledge of the spatial and temporal dimensions of hydrosystem dynamics.

## Coupling the metaecosystem framework and the ecosystem service chain to understand ecosystem services maintenance and delivery in sociohydrosystems

Ecosystem services are divided into three categories: provisioning, regulation and maintenance, and cultural services (Haines-Young and Potschin 2018). Ecosystem services are regulated by biophysical structures and ecosystem processes (the cascade model; Haines-Young and Potschin 2010) operating over multiple spatiotemporal scales and sensitive to human activities (Truchy et al. 2015). For instance, regulation and maintenance services include various ecosystem-level processes involved in the cycling of nutrients and energy (e.g., organic matter decomposition, nutrient cycling), the generation and maintenance of ecosystem structures (e.g., soil formation, channel morphology), or the maintenance of populations (Smith et al. 2013).

**Figure 2.**
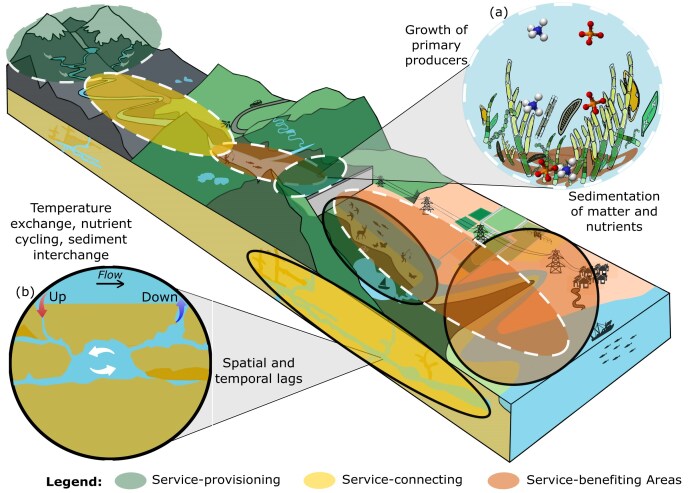
Illustration of the service provisioning chain including the service-provisioning, service-connecting, and service-benefiting areas in the spatiotemporal template of sociohydrosystems. Two ecosystem services (CICES v5.2) are represented on the figure: “Regulation of the chemical condition of macronutrients” (the dashed ellipses) (a) and “Regulation of chemical composition and temperature” (the solid ellipses). Nutrients, organic matter and sediments originate from headwaters which acts as service-provisioning areas (the dashed green ellipse) and travel downstream through the stream network which acts as service-connecting area (dashed yellow ellipse) to end up in a downstream lake which acts as service-benefiting area (the dashed orange ellipse), boosting lake productivity. However, the lake also acts as a service provisioning area (service-provisioning area; the small dashed green overlapping the orange dashed ellipse) as the received organic matter and nutrients are trapped in lake sediments and used by lake primary producers, therefore protecting downstream waters (service-benefiting area) from eutrophication (the dashed orange ellipse below the dam). In parallel, vertical transfers of surface water contribute to the regulation of chemical composition and temperature of the environment (b). Surface water (the solid green ellipse) infiltrates through fissured rocks or karstic grounds and transits through groundwaters (service-provisioning and service-connecting areas; the solid yellow ellipses) for a certain time and distance, ultimately being released in downstream river sections (service-benefiting area; the solid orange ellipse), creating spatial and temporal lags.

Applying the service provision chain to sociohydrosystems (figure 1a; Fisher et al. 2009) brings in spatiotemporal realism and allows to bridge the gap with metaecosystem approaches. In sociohydrosystems, the river and its surroundings represent the main service-connecting area of many ecosystem services (figure 2). For example, the provision of drinking water is sustained by groundwater tables and water flows through rivers and streams from the upstream part of the sociohydrosystems (headwaters; service-provisioning areas) to downstream cities (service-benefiting areas). In addition, time lags between service-provisioning and service-benefiting areas can be expected, depending on the service-connecting areas' properties and conditioned by the sociohydrosystems template. In fact, in many cases, as is illustrated by the rich literature about the relationships between biodiversity and ecosystem functioning (van der Plas 2019), the underlying function, rather than the service itself, is spatialized or timed by the sociohydrosystems characteristics (but see Le Provost et al. 2023). We illustrate how the service provision chain can be associated with the different categories of organism movements and passive flows of resources following a metaecosystem organization in sociohydrosystems.

### *Ecosystem* services driven by organism movements

We first start by describing organism movement that drive ecosystem services.

#### Foraging movements

Foraging involves short-distance and frequent animal movements, conveying organic matter across habitats or ecosystems (Subalusky and Post 2019) and playing a crucial role in underlying ecosystem functioning. Reaching out for water daily is common for many terrestrial vertebrates, and therefore, sociohydrosystems constitute a feeding habitat for many species. Sociohydrosystems therefore represent daily crossroads of organisms and matter, which, in turn, sustain many crucial ecosystem services depending on population maintenance, nutrient cycling, and recreational activities.

The foraging activities of beavers bringing riparian trees into sociohydrosystems is an example of a metaecosystem process influencing ecosystem services delivery (Thompson et al. 2021). The beavers' engineering effects on sociohydrosystems through dam construction affect stream water level and flow velocity by flooding areas, which, in turn, deliver several ecosystem services, such as flood regulation, water purification, water supply, carbon sequestration, and habitat and biodiversity provision (Thompson et al. 2021). Another classic example is grazing hippopotamuses on riparian grasslands that also defecate in the water (figure 1b; Subalusky et al. 2015), bringing enormous amounts of organic matter into the aquatic systems. This organic matter subsequently sustains carbon and nutrient cycles (Subalusky et al. 2015) and indirectly brings important economic and recreational activities (e.g., ecotourism; Bonye et al. 2022). Together, these two examples show that, considering foraging movements, sociohydrosystems can act as service-benefiting areas for recreational and economic activities, as service-provisioning areas for nutrient cycling, and often as service-connecting areas for water supply.

#### Seasonal migration

Seasonal migrations are the predictable temporal pulses of organisms connecting ecosystems across spatial scales (Bauer and Hoye 2014). This spatiotemporal coupling among distinct geographic areas (henceforth, *telecoupling*; Liu et al. 2013) acknowledges that migrations play a crucial role in ecosystem services delivery (Bauer and Hoye 2014) at a given place and time (service-benefiting areas). This delivery depends on subsidies and ecological processes in similar or distinct habitats, sometimes kilometers away from each other (service-provisioning areas; Liu et al. 2020). This telecoupling requires preserving ecosystem services at a given place but also distant habitats and migratory routes (service-connecting area; Liu et al. 2020). In sociohydrosystems, seasonal migrations can be related to mass transfer of insects and fish between aquatic habitats (Pennuto et al. 2021, Srayko et al. 2022) or of organisms between terrestrial and aquatic systems (Bauer and Hoye 2014, Cayuela et al. 2020).

For example, in autumn, thousands of boatmen (Hemiptera) migrate from small isolated wetlands (service-provisioning areas) to large rivers (service-benefiting areas) in the Prairie Pothole Region of North America, where they sustain the food web by becoming the main food source for several riverine fish (e.g., goldeye and mooneye) before winter (Srayko et al. 2022). Ultimately, this seasonal migration sustains both recreational and professional fishing. Seasonal migrations can also occur among different habitats within the same ecosystem. For instance, round gobies, a lacustrine species, perform seasonal offshore nearshore migrations in Lake Ontario (which spans the United States and Canada; Pennuto et al. 2021). The late autumn offshore migration represents a major food source for lake sturgeons in winter. Sturgeon populations in the Great Lakes region in North America are highly valued by professional fisheries (i.e., for caviar) and culturally and spiritually by First Nations communities (Ontario Ministry of Natural Resources 2009). Similarly, seasonal waterbird migrations connect intercontinental sociohydrosystems (e.g., arctic terns migrating from Antarctica to Iceland for breeding; figure 1c) and sustain, for example, seasonal bird watching tourism (Liftenegger et al. 2014).

#### Ontogenetic migration

Ontogenetic migrations represent another type of organism movements with crucial implications for many ecosystem services in sociohydrosystems. During ontogeny, drastic physiological changes, habitat shifts and cross-ecosystem flows of organisms and matter may occur (Gounand et al. 2018a). For example, ontogenetic migrations of insects (e.g., emergence) between aquatic and terrestrial habitats; spillover effects (Scherer-Lorenzen et al. 2022) often play an important role in, for example, pest control of adjacent crops, in what are considered service-benefiting areas by farmers. Indeed, emergent aquatic insects sustain the community of predators such as terrestrial spiders during periods characterized by low abundances of crop insect pest (Raitif et al. 2019). The emergence of aquatic larvae into flying adults also contribute to pollinating both wild and cultivated plants, which not only affect plant metacommunity dynamics but also ensure food production—a crucial ecosystem service (Raitif et al. 2019).

Ontogenetic migrations of some fish populations is another example of ecosystem boundary crossing that sustains provisioning, regulation and maintenance, and cultural ecosystem services (Villamagna et al. 2014). Salmonids realize two ontogenetic migrations, from their riverine birthplace to their growth place in the sea (both the river and the sea are considered service-provisioning areas for early fish developmental stages) and then from the sea to their birth rivers for reproduction (figure 1c). The second migration occurs when individuals reach a sufficient weight to be fished, and lower stretches of the river represent a service-connecting area, whereas upstream reaches of the river become a service-benefiting area for anglers and tourists enjoying the spectacle of bears fishing salmonids (Penteriani et al. 2017). During migration events, salmonids also help dispersing riparian plants (Hocking and Reynolds 2011), transferring high-quality marine nutrients in headwaters and adjacent terrestrial forest (salmonid carcasses brought by bears) that sustain ecosystem functioning (Holtgrieve and Schindler 2011). In this example, the same element of the sociohydrosystems, river or sea, can be considered successively as a service-connecting, a service-benefiting area, and a service-provisioning area, depending on the stage of the fish life cycle. Beyond their role as corridors bringing fishes to the service-benefiting area, service-connecting areas can directly influence the provision and value of associated ecosystem services. The service-connecting area structural connectivity (i.e., dams and weirs in the watershed; e.g., Brevé et al. 2014) is a key driver of salmonid movements, both in terms of ontogenetic migration and gene exchanges (through dispersal) at the metapopulation scale (Perrier et al. 2011). Moreover, the service-connecting area physicochemical characteristics (e.g., water temperature, turbidity, nutrients concentrations) coupled with hydrodynamic factors (e.g., alternation of lentic and lotic habitats, oceanic currents) influence the fish body condition and growth rate (Utne et al. 2021) and, therefore, the timing of the associated ecosystem services delivery in service-benefiting areas.

#### Dispersal

Dispersal is an organism movement associated with reproduction, linking locations that belong to the same ecosystem type, with or without a crossover between multiple ecosystems (Gounand et al. 2018a). For a migratory salmon, dispersal could occur between different tributaries within a watershed or between different tributaries from different watersheds. In this case, rivers play the multiple roles of service-provisioning, service-connecting, and service-benefiting areas from an angler point of view. In a metapopulation context, reproducers incoming in previously isolated populations drive population persistence through rescue effects (Granzotti et al. 2021) and maintain or enhance already existing ecosystem services (e.g., fishing, sightseeing, food production). Dispersal allows the colonization of new territories or the recolonization of temporally unoccupied habitats, potentially enhancing the already existing ecosystem services or bringing new ecosystem services.

Rivers are also service-connecting area that ensure the passive dispersal of plant seeds and propagules (figure 1e) and provide environmental conditions favorable for their establishment along the riverbanks (Nilsson et al. 1989). In turn, riparian vegetation attracts a riparian-specific biodiversity (e.g., birds, flying insects and flowers) and is considered as service-benefiting areas for naturalists, because it contributes to recreation and ecotourism activities (Riis et al. 2020). Distant sociohydrosystems can also be connected through dispersal of propagules and small organisms through their hosts’ movements that undergo seasonal, ontogenetic migrations or foraging movements themselves (Gounand et al. 2018a). For instance, waterbirds can act as major vectors of long-distance dispersal of aquatic plants and invertebrates in recipient sociohydrosystems (Green et al. 2023).

### Ecosystem services driven by flows of energy and matter

Flows of matter (nutrients, organic matter, pollutants) occur both inside the hydrological network and between aquatic and terrestrial habitats that constitute reciprocal resource subsidies for metacommunity dynamics (Nakano and Murakami 2001, Cole et al. 2020) and ecosystem functioning of both habitats (Scherer-Lorenzen et al. 2022). These flows are driven by both abiotic and biotic factors (Larsen et al. 2016, Nakano and Murakami 2021) and are at the origin of numerous ecosystem services (e.g., water purification, retention of contaminants and excessive nutrients). The characteristics of subsidies depend only on the habitat that constitutes the service-provisioning area and would strongly influence the biota that benefits from subsidies.

#### Flows of nutrients and organic matter

Flows of organic matter and nutrients among marine, aquatic, and terrestrial habitats are key drivers of community dynamics (Gounand et al. 2018a, Montagano et al. 2019). In river ecosystems, both terrestrial and aquatic habitats can be either a service-connecting area or a service-benefiting area, depending on the nature of the lateral (i.e., across ecosystems) or longitudinal (along the river network or riparian corridors) flows (Torgersen et al. 2022). For instance, riparian trees are service-provisioning areas when arboreal arthropods along with leaf litter fall (arthropod rain) into the stream—the service-benefiting area—providing high-quality resources for aquatic organisms (Carassou et al. 2017), influencing community assemblages and trophic cascades within the aquatic food web, sustaining fish growth (Cole et al. 2020), and associated ecosystem services (e.g., recreational fisheries). Conversely, riparian habitats become service-benefiting areas when enriched in nitrogen and phosphorus by, for example, the mass mortality of adult insects emerging from their aquatic habitats (Raitif et al. 2019).

Organic matter and nutrients originating from headwaters' (service-provisioning areas) runoff, precipitation, and organism movements travel through the stream network to downstream lakes and reservoirs, ultimately influencing downstream ecosystem services (service-benefiting areas; figure 2a; see Brett et al. 2017 for positive effects on the service-benefiting areas and Dutton et al. 2018 for detrimental effects). However, through the retention and sedimentation of matter and nutrients, connected wetlands also act as service-provisioning areas, because they ensure buffering effects that prevent all these compounds from reaching downstream waters (service-benefiting area), protecting them from excessive sediment loads and eutrophication (figure 2a; Heino et al. 2021). Finally, organic and nutrient exchanges occur at the river–sea interface, with nitrogen and carbon transported from the sea (service-provisioning areas) by migratory fish (Poulet et al. 2022), ultimately sustaining river biota (service-benefiting area; Wipfli and Baxter 2010) and ecosystem services associated with river fish production.

#### Flows of energy and inorganic matter

Water quantity, temperature, and quality have profound impacts on community maintenance and the underlying ecosystem processes in service-provisioning, service-connecting, and service-benefiting areas. Vertical transfers of water to and from groundwaters contribute to temperature exchange, nutrient cycling, and sediment interchange between surface waters and underground ecosystems (figure 2b; Sacco et al. 2024). Waters from the surface (service-provisioning area) can therefore stay for some time in groundwaters (service-connecting area), leading to both spatial and temporal lags between surface service-provisioning and service-benefiting areas (figure 2b). This residence time allows for a more regular supply of thermally buffered water to the surface throughout the year (Caldwell et al. 2020). Temperature buffering and reduction of insolation levels can also result from riparian shading, providing suitable thermal conditions for biota (Cole et al. 2020).

Rainwater runoff erodes agricultural lowlands (service-provisioning areas; Montgomery 2007), and the resulting sediments are carried through the stream network (service-connecting area) to river deltas and estuaries (service-benefiting areas), where they will form sandbars and mudflats (Besset et al. 2019). Water currents shape sandbars' and mudflats' topography, ultimately influencing, for example, biodiversity establishment and recreational ecosystem services such as sunbathing and boating (Defeo et al. 2009).

## Implications of an ecosystem services metaecosystem approach for sociohydrosystem management

Most current management and restoration projects of sociohydrosystems only address local-scale processes within individual ecosystems, whereas focusing on ecosystem services calls for integrating large spatial scales and cross-ecosystem interactions (Tolvanen and Aronson 2016, Friberg et al. 2017). For example, to preserve valuable ecosystem services such as recreation (e.g., swimming, fishing, sightseeing), irrigation, and drinking water supply, lake restoration from eutrophication consists of in-lake treatments that have positive but short-term effects on lake characteristics (Søndergaard et al. 2010, Kong et al. 2023). On the contrary, broadscale management measures aiming at reducing the nutrient loads from the catchment area, including inputs from nonpoint sources, show persistent positive effects (Nakhaei et al. 2021). It is therefore clear that the long-term success of managing sociohydrosystems from both an ecosystem services and metaecosystem perspective is more stable and robust over time if the processes affecting the disturbances are apprehended at adequate spatiotemporal scales, which may extend beyond the management area and sociohydrosystems themselves.

An ecosystem services metaecosystem approach is currently missing in environmental policies and legislations. The very first legislation and management measures focused on preserving pristine nature (i.e., nature for itself), with an emphasis on conserving terrestrial areas with high taxonomic diversity (i.e., high species richness, endemic and charismatic species; Prip 2018) but having little effect on aquatic ecosystems (Herbert et al. 2010). With the Water Framework Directive (WFD 2000, Hermoso et al. 2016), the goals then shifted toward mitigating anthropogenic impacts occurring at broader spatial scales, such as diffuse water pollution, the effects of deforestation, or the impacts of hydropower dams (e.g., Truchy et al. 2022). This first shift in management policies to address the environmental issues at larger scales embraced the concepts of metaecosystem (Schiesari et al. 2019) but not ecosystem services. Ecosystem services' inclusion in management policies helps evaluate management efforts combining both ecological goals and socioeconomic demands (Friberg et al. 2017), in line with the rising perspective of a shared human–nature environment (i.e., people and nature; Hermoso et al. 2016).

Including ecosystem services and their spatial components (service-provisioning, service-connecting, and service-benefiting areas; Fisher et al. 2009) brings in an additional complexity when designing management measures, because the spatial scale of the ecosystem services range to be considered does not necessarily coincide with the spatial scale of the ecological processes and conservation goals targeted by management plans (e.g., Wilbanks 2006). In addition, such spatial mismatch between ecosystem services and management plans may collide with administrative boundaries. Below, we provide three examples of approaches showing how specific, concrete actions can be taken locally and can have impacts at the metascale, and their potential implications for ecosystem services bundles—sets of ecosystem services that co-occur at a specific location and at a particular time (Saidi and Spray 2018).

### Cascade model approach for efficient local hydrosystem restoration

Considering and assessing ecosystem services can be helpful in making decisions for restoration projects. A way of representing the logic behind ecosystem services is to apply Haines-Young and Potschin’s (2010) cascade model. In this model, ecosystems are defined by properties (i.e., biophysical structures and ecosystem processes) producing ecosystem functions. These functions provide benefits for the society to which a value, sometimes monetary, can be attributed. Ecosystem services include the supply (i.e., function) and the demand (i.e., benefit). Management, restoration, and policy actions can be made to limit the anthropogenic impacts affecting ecosystem properties, in order to sustain ecosystem services delivery and vice versa. Considering ecosystem services could guide decision-making when designing management actions and restoration projects.

To investigate how local restoration actions may enhance ecosystem services at larger spatial scales, we provide evidence from a case study of the tidal restoration of a marsh in the largest macrotidal estuary in Europe, the Gironde estuary (Rambonilaza et al. 2021, Carassou and Vildier 2022). Nekton community (i.e., communities formed by fishes and macrocrustaceans able to actively disperse in aquatic ecosystems) responses to the restoration of tidal connectivity between the marsh and the estuary were compared with nekton community responses from dyked marshes in the estuary (Lechêne et al. 2018). Following the tidal restoration, a rapid recovery of estuarine and marine-related fish communities in the marsh was observed (Lechêne et al. 2018). Communities from tidally restored marshes were later shown to support functional aquatic food webs involving typical estuarine and marine consumers, whereas marshes in which the tidal connectivity remains restricted mostly support freshwater-type food webs dominated by introduced species from the nekton (Carassou and Vildier 2022). Economic benefits arising from the tidal restoration of these marsh habitats were estimated on the basis of the cascade model (Haines-Young and Potschin 2010) and upscaled to the whole estuary. They were noticeably positive for the professional fishery, recreational fishery, and consumers of seabass (Rambonilaza et al. 2021). This case study therefore illustrates how restoration efforts undertaken on specific habitats and at local scale may enable a cumulative delivery of ecosystem services at much larger spatiotemporal scales, through the spillover effect.

### Nature-based solutions for mitigating water pollution and protecting drinking water

Some nature-based solutions and ecological engineering involve the use of plant materials to reduce flows of organic and inorganic matter which can be considered as disservices (Blanco et al. 2019) and therefore compromise other ecosystem services, such as drinking water supply or recreational activities. These management practices, already in place in anthropized ecosystems such as agroecosystems, take into account the flows of organisms and matter described in metaecosystem theories (McCann et al. 2021). For instance, buffer zones (Valkama et al. 2019) and constructed wetlands (Martínez-Espinosa et al. 2021) in agroecosystems aim to reduce nitrogen leaching from crop areas to the surrounding aquatic habitats to avoid disservices associated to eutrophication (Burgin et al. 2013). Vegetation development is another example of active erosion and sedimentation control of gullies (Frankl et al. 2021). The use of bioengineering structures, such as brush layers on wooden sills, has indeed been found to enhance sediment trapping during water flow. Accumulated sediments contribute to plant resprouting and survival in gullies, leading to increased ecosystem services such as sediment trapping during subsequent runoff events and habitat creation and maintenance.

### Portfolio theory for optimising ecosystem services: The case of Pacific salmon

In economics, portfolio theory (Markowitz 1952) is used to optimise investments in a diversity of assets (e.g., commodities) such that they achieve a balance between financial gain and investment risk. Applied to ecology, genes, populations, species, or ecosystems can be considered assets (Figge 2004). Portfolio theory therefore provides a framework for characterizing the relative performance in ecosystem services delivery (e.g., brown trout for recreational fishing) among different assets (e.g., different populations of trout) that could display a diversity of responses in space and time (e.g., because of local adaptation). Some ecological studies nowadays apply portfolio theory to guide better ecosystem management practices.

**Figure 3.**
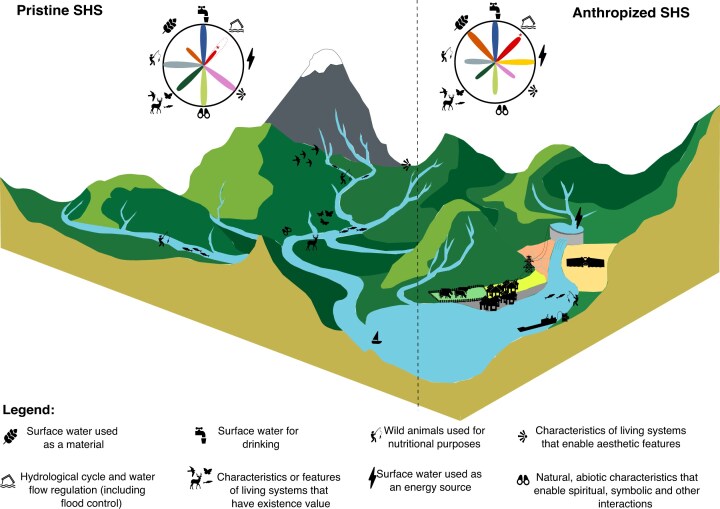
Ecosystem service bundles, synergies, and trade-offs. Eight ecosystem services are exemplified, with their descriptions extracted from the CICES v5.2 (Haines-Young and Potschin 2018). The petal diagrams present ecosystem services bundles—that is, sets of ecosystem services that co-occur within a given watershed at a particular time. The size of the petal ranges between 0 and 1 and reflects transformed effect sizes of statistical models, the outer circle representing the maximum value of 1 (Gamfeldt et al. 2013). The size of the petal of the ecosystem services flood control (in red with an asterisk) is still controversial (Ma et al. 2022) and can therefore either be small or big (the red-dashed big petal in pristine sociohydrosystems and the black dashed small petal in anthropized sociohydrosystems). Some ecosystem services are in synergy (i.e., positive coefficient of correlation, Gamfeldt et al. 2013; e.g., recreational fishing and biodiversity), whereas others present trade-offs (i.e., negative coefficient of correlation, Gamfeldt et al. 2013; e.g., water for irrigation and wildlife watching). Synergies and trade-offs among ecosystem services can change through time and depend on the sociohydrosystem context (pristine versus anthropized).

Griffiths and colleagues (2014) investigated Pacific salmon return in various Canadian sociohydrosystems. Salmon return can be seen as a proxy of several ecosystem services, including recreational fishing, aesthetic, cultural heritage, and food security. Using metapopulation theory, they created portfolios including different sites at various spatial scales (local to regional). They assessed the variability of return within and between populations to evaluate the performance and reliability of salmon fisheries in sociohydrosystems over time. They showed that salmon portfolios in near-pristine sociohydrosystems (i.e., intact habitat geographically distant from the ocean) were more reliable than the ones from human-affected sociohydrosystems (i.e., presence of numerous dams and a high human footprint, see figure 3 for examples of pristine and anthropized sociohydrosystems). Pristine habitats also produced a diversity of responses among sociohydrosystems (e.g., asynchronous return due to a diversity of life history among populations) that reduced covariance in salmon return and favored high-performing portfolios. In other words, poor salmon return in several populations is compensated for by other well-performing populations, leading to an overall reliable portfolio. In contrast, anthropogenic drivers may synchronise populations (e.g., the loss of specific habitats and associated populations, habitat homogenization reducing response diversity, reduced genetic diversity due to hatcheries), leading to poor-performing portfolios (i.e., high covariance in salmon return among populations).

After assessing the portfolios, management actions can be tailored to optimize ecosystem services' delivery associated with salmon return (Moore et al. 2021). In portfolios where synchronous return among populations is the greatest risk, managers may focus on maintaining or restoring disturbance regimes that promote response diversity and decrease population synchrony. On the contrary, in portfolios where asynchronous return is the greatest risk, management measures may explore whether within-population response diversity may be enhanced through increasing habitat heterogeneity or maintaining the full life history diversity of the population. So far, portfolio theory is mostly focused on the metapopulation level when optimizing the trade-offs between fishery productivity and management strategies (i.e., mixed-stocked fishery; Moore et al. 2021), ensuring reliable ecosystem services delivery. However, portfolio theory can also offer a huge potential in managing resources in the multispecies context of a metacommunity by considering the species richness and diversity of community responses. In addition, portfolio theory represents an interesting tool when addressing a single and well-defined ecosystem service, but its full potential is yet to be harnessed when handling a bundle of ecosystem services involving multiple ecosystem services trade-offs and various processes acting across spatial, temporal, and hierarchical levels.

### Ecosystem services trade-offs and synergies

Ecosystem services usually occur in bundles, and within these bundles, interactions among the different ecosystem services exist according to trade-offs and synergies. Ecosystem services trade-offs arise when the provision of one ecosystem service is enhanced at the cost of the production of another ecosystem service, whereas ecosystem services synergies arise when several ecosystem services are enhanced simultaneously (Bennett et al. 2009, 2015). The type and strength of these ecosystem services interactions may vary over time and space (Queiroz et al. 2015, Lindborg et al. 2017). For instance, salmon returns sustain an ecosystem services bundle (usually represented as a petal diagram) in which professional and recreational fisheries are enhanced, along with bioremediation and regulation of the chemical conditions (for quality of drinking water and nutrient cycles; figure 3). Other societal demands, including hydropower production and flood regulation, impede the provision of ecosystem services associated with salmon returns by breaking longitudinal connectivity of the river network because of damming (figure 3; Brevé et al. 2014).

Understanding the complex relationships within ecosystem services bundles that may have different spatiotemporal dynamics is of high importance to generate better outcomes for human societies (Tallis et al. 2008, Mitchell et al. 2015) and for future decision-making processes (Piccolo et al. 2019, Torres et al. 2021). Indeed, ecosystem services bundles may arise from the ability of sociohydrosystems to perform multiple processes (Raudsepp-Hearne et al. 2010) but also from the existing connectivity between aquatic ecosystems (freshwater, estuarine, and marine) and the adjacent aquatic or terrestrial ecosystems. For instance, freshwaters are especially valued for the provision of drinking water and recreation activities such as sightseeing, fishing, and bathing, which all require good water quality. Crops and fiber production in adjacent terrestrial habitats may not be of interest for freshwater managers but they nevertheless undermine the delivery of freshwater ecosystem services, because of nutrient runoff, pesticide spillover, and water abstraction. In this context, managers need to consider both the meta approach and the spatiotemporal dynamics of ecosystem services bundles in order to enhance the connectivities between sociohydrosystems and other ecosystems (e.g., Friberg et al. 2017) that favor desirable ecosystem services while reducing other connectivities to limit disservices (e.g., spread of environmental pollution, Tsygankov et al. 2022; waterbird-mediated dispersal of invasive red-water ferns, Reynolds et al. 2015).

## Future research directions for developing a metasystem approach for ecosystem services management

Although the conceptual framework of metaecosystems is now abundantly developed and can nourish ecosystem services thinking, there is an open avenue for further research directions to address the remaining challenges when using meta approaches for ecosystem services management in sociohydrosystems. These challenges concern conceptual, methodological, and epistemological developments.

### Conceptual challenges

One of the biggest conceptual challenges with respect to developing a meta approach of ecosystem services in sociohydrosystems is to understand and resolve the causal chain linking metaecosystem processes with services. For instance, this may be achieved by modeling the ecological functions or traits involved in the chain (as proposed for fish in Villéger et al. 2017 or applied on grassland plants in, e.g., Waldén et al. 2023). Some interactive attempts have also been proposed to better understand and visualise these causal chains—for example, for river restoration (project WISER; www.wiser.eu/results/conceptual-models) with models depicting the links between the restoration action, the abiotic and biotic compartments and the consequences in terms of ecosystem function and service (e.g., water quality). Such a conceptual framework could be enriched by integrating a process-based metaecosystem approach (Gounand et al. 2018a), to achieve a finer understanding of spatiotemporal dynamics in ecosystem services distribution in sociohydrosystems (Harvey et al. 2023).

A second important challenge is to investigate the role of time and timescales into ecosystem services distribution in sociohydrosystems. For instance, metacommunity ecology has experienced its paradigm shift by acknowledging the importance of considering community temporal dynamics to unravel the role of different metacommunity processes in biodiversity distribution (e.g., Jabot et al. 2020, Guzman et al. 2022). Likewise, we need to clarify how time modulates ecosystem services provision chain dynamics, in particular how a service at a given time may become a disservice at another time or how the service-benefiting, service-connecting, or service-provisioning nature of a sociohydrosystem varies depending on the rhythm of seasons, hydrodynamics, decadal cycles, or long-term changes (e.g., climate change). It is also crucial to anticipate the potential spatiotemporal mismatches between the societal demand and the ecosystem services provision (see, e.g., Winkler et al. 2021). Identifying the causes of such spatiotemporal mismatches is determinant to ensure ecosystem services maintenance. This requires assessing ecosystem properties with adequate metrics. For instance, in the context of a biodiversity crisis including accelerated extinction rates and rapid species turnover, monitoring biodiversity accounting for variation in abundance and population genetic diversity is crucially needed to account for the time lag associated with extinction debt that is easily overlooked by presence or absence biodiversity metrics, but of the utmost importance to anticipate population extirpation and possibly threat to ecosystem services maintenance.

A third challenge lies in our abilities to predict the effects of abrupt changes on ecosystem services as ecosystems are driven by a complex interplay between long trends and short extreme events (e.g., drought, flood, pest emergence) embedded in a multiple-stressors context (Rocha et al. 2018, Jackson et al. 2021, Turner et al. 2020). The increasing frequency of extreme events of different nature can lead to a cascading regime shift (i.e., tipping points) across ecosystems (Turner et al. 2020) involving drastic changes in environmental conditions (e.g., from oligotrophic to eutrophic) with the potential to severely alter ecosystem services (e.g., global warming and flow intermittency increase organic matter accumulation and alter carbon sequestration; Pérez-Silos et al. 2025). However, temporal occurrences of such abrupt changes remains difficult to predict because the frequency of stressor events can drastically influence the ecological memory of an ecosystem (i.e., the ability of past stressors to influence the future ecological responses of a population, community, or ecosystem, Jackson et al. 2021) and ultimately govern the supply of ecosystem services (Johnstone et al. 2016).

A fourth challenge is to consolidate the conceptual foundations of the bundle view of metaecosystem services and to better understand the complex relationships between multiple ecosystem services (Tallis et al. 2008, Bennett et al. 2009). This will require considerably broadening the ecosystem functions that are simultaneously monitored, so that their covariance structure and therefore the trade-offs involved within ecosystem services bundles (Howe et al. 2014, Bennett et al. 2015) can be understood. Such simultaneous monitoring calls for more integrated and large research initiatives to associate the necessary skills and expertise (e.g., Datry et al. 2021). Understanding these complex relationships is of primary importance to maximise the provision of ecosystem services in a given place and time (Waldén et al. 2023).

Tackling these challenges will definitely require bridging the gap among metaecosystem, landscape, and ecosystem services approaches across administrative boundaries associated with management planning. Indeed, the metaecosystem framework allows the development of a more integrative perspective on fluxes and organismal movements but with a focus between ecosystems. Adopting a landscape perspective is needed to encompass the range of spatial and temporal scales of fluxes between and within ecosystems (Gounand et al. 2018a). As such, integrating fluxes between terrestrial and aquatic ecosystems with a riverscape approach (Torgersen et al. 2022) would allow us to track more thoroughly the dynamics of the fluxes shaping the dynamics of the ecosystem services in sociohydrosystems (Harvey et al. 2023).

### Methodological and technical challenges

Although noticeable progress is made with the conceptualization of the links between metaecosystem dynamics and ecosystem services spatiotemporal distribution, data collection on spatial and temporal ecological fluxes and on their impacts on ecosystem services is still in delay (Gounand et al. 2018c, Talluto et al. 2024). Ecologists may now widely engage in developing spatiotemporal monitoring of ecosystem fluxes to document spatial couplings that are often temporally variable. In this respect, continuous (and automatic) monitoring methods are optimally suited to detect sudden events (Moiroux-Arvis et al. 2023) that may have an overwhelming weight on cumulative outcomes for ecosystem services (e.g., phosphorus leaching during floods, e.g., Macrae et al. 2011; rewetting events and organic material pulses, e.g., Von Schiller et al. 2019). In addition, remote-sensing methods nowadays allow monitoring sediment flows and riparian change through time (e.g., Piégay et al. 2020). Finally, DNA monitoring has the potential to substantially contribute to the intensive monitoring of biodiversity, including intraspecific genetic diversity, in sociohydrosystems (Carraro et al. 2020, Andres et al. 2023) and to investigate its role in the spatiotemporal diffusion of ecosystem services along whole river systems.

A second methodological challenge ahead is to develop versatile modeling methods to upscale monitored fluxes at the appropriate scale for ecosystem services diagnosis. Geospatial approaches (such as geographical information systems) offer a first step in this direction by enabling computations of ecosystem services from land cover data (e.g., water quality; Topp et al. 2020). However, such approaches need to be complemented with more sophisticated ones (e.g., Chalkiadakis et al. 2022 for marine ecosystems), especially those involving community dynamic models figuring flows of organisms and matter across land covers. For instance, fluxes may be driven by a complex interplay between species complementarity or sampling effects and landscape context (Mitchell et al. 2024), thereby requiring mechanistic modeling approaches (Ellis-Soto et al. 2021).

A third technical challenge falling within the scope of biodiversity–ecosystem service research (e.g., Waldén et al. 2023) is to be able to quantify the relative contribution and spatiotemporal dynamics of different metaecosystem processes in the distribution of given ecosystem services. Being able to produce such quantitative assessments would allow identifying the processes (e.g., organismal movements and matter and energy fluxes) that need to be stimulated to restore or maintain a given ecosystem service's distribution in a given context. To address this question, one could use a synthesis approach (Halpern et al. 2023) by analysing multiple empirical data sets from studies that address the links between a given metaecosystem process (e.g., water flow) and an ecosystem services product (e.g., drinking water).

Finally, the service-provisioning, service-connecting, and service-benefiting area framework described throughout this article is mainly focused on a single ecosystem service. Working with multiple ecosystem services requires defining spatial and temporal dynamics of the service-provisioning, service-connecting, and service-benefiting areas of each ecosystem service. Current approaches focus on service-provisioning or service-benefiting areas of each ecosystem service separately in an additive way, often ignoring fluxes (service-connecting area, but see InvEST for taking into account physicochemical fluxes). However, the service-provisioning, service-connecting, service-benefiting area framework remains useful for managers as they help to map areas—and their future projections—that need to be prioritised in terms of conservation or restoration (Ball et al. 2009, Schröter and Remme, 2016), to be aware of area overlaps when working within a multiple ecosystem services context, and to conceptualize the flows between service-provisioning and service-benefiting areas by incorporating metatheories. We acknowledge that addressing independently the service-provisioning, service-benefiting, and service-connecting areas of each ecosystem service in an additive framework as currently proposed is a first step toward further developments accounting for the multidimensional covariation among ecosystem services. Such a challenge could be addressed through the design of a multivariate version of service-provisioning, service-connecting, and service-benefiting areas taking into account the synergies and trade-offs among ecosystem services relevant for a large range of stakeholders.

### Epistemological challenges

Overall, research on the spatiotemporal distribution of ecosystem services needs integrative and interdisciplinary approaches (Raymond et al. 2013, Fernandez et al. 2022). Bringing metatheories into this research is key, because it will help evaluate the consequences of matter and energy fluxes and of biodiversity changes at the landscape scale, supporting the heterogeneous distribution of services. This research will also have to feed on and collaborate with research on externalities, to list and investigate services at a given location (service-provisioning area) supplying a remote service-benefiting area from the prism of socioeconomic and cultural values and environmental footprint (Chan et al. 2012, Bellver-Domingo et al. 2016, Bellanger et al. 2021).

To go a step further and strengthen the link between the ecological and social components of the sociohydrosystem (Dunham et al. 2018), we could approach both of them through the prism of meta. Although anthropogenic impacts on metaecosystem dynamics are already taken into account when quantifying flows (Gounand et al. 2018b), one could consider using a metaecosystem approach to apprehend sociosystems and their functioning (Renaud et al. 2018). In such a metasocioecosystem, socioecological mechanisms are included to apprehend the impacts of human societies on the flows connecting ecosystems. Indeed, a service produced by a given group of people in a given place and time could benefit several social levels of organization (similar to levels of biological organisation) across other spatial and temporal scales. For instance, the restoration of a riparian forest, although mainly targeting the recovery of nutrient cycling, bank stabilization and recreational services for the local population, may be part of a wider national strategy of forest restoration and contribute more generally at national and international levels to carbon dioxide capture, climate regulation, and biodiversity conservation. Likewise, human fluxes (along a service-connecting area or through a service access area—typically, transportation networks; Antognelli et al. 2018) connect service-provisioning areas, service-benefiting areas, and areas where ecosystem services can be consumed, potentially reshuffling ecosystem services distribution. This is the case for shipping that could ensure transports of goods and people but could also be a vector of invasive species threatening service-benefiting areas (e.g., Keller et al. 2011, Karatayev and Burlakova, 2022), while justifying continuous hydromorphological recalibration of rivers and estuaries.

Ecosystem management ultimately leads to making societal and political choices and considering ecosystem services is no exception. Ecosystem services trade-offs are common and maximizing multiple co-occurring ecosystem services may be challenging as contradicting interests of different stakeholders have to be balanced out (Howe et al. 2014, Bennett et al. 2015). Applying a metasocioecosystem approach by quantifying socioecological fluxes could help anticipate disservices, inform mitigation measures, and ultimately provide crucial insights for decision-makers (Brauman et al. 2007).

## Conclusions

By linking metaecosystem and ecosystem services theories, we propose a unified framework for understanding and managing ecosystem service delivery in sociohydrosystems. Our framework uses metaecosystem processes, based on biotic and abiotic flows, and links it to a more integrative landscape approach. This merging should allow for a better understanding of the effects of barriers, corridors, and sources of fluxes onto the delivery of ecosystem services within a complex landscape. In particular, we believe that taking into account ecosystem bundles is crucial in order to design international management strategies for multiple ecosystem services, therefore avoiding unbalanced ecosystem service delivery and in fine social inequalities (Villarreal-Rosas et al. 2022). Although it will improve our understanding of spatial dynamics and their consequences for ecosystem services, incorporating temporal variability remains challenging, especially within the context of global change bringing in new environmental conditions (e.g., new and long drying events, intense flooding, changes in hydrological cycles, glacier melting, increase in marine levels).
